# Geographical variations and influential factors in prevalence of cardiometabolic diseases in South Korea

**DOI:** 10.1371/journal.pone.0205005

**Published:** 2018-10-02

**Authors:** Won Seob Oh, Sanghyun Yoon, Juhwan Noh, Jungwoo Sohn, Changsoo Kim, Joon Heo

**Affiliations:** 1 School of Civil and Environmental Engineering, College of Engineering, Yonsei University, Seodaemun-gu, Seoul, Korea; 2 Department of Preventive Medicine, College of Medicine, Yonsei University, Seodaemun-gu, Seoul, Korea; Erasmus MC, NETHERLANDS

## Abstract

Geographical variations and influential factors of disease prevalence are crucial information enabling optimal allocation of limited medical resources and prioritization of appropriate treatments for each regional unit. The purpose of this study was to explore the geographical variations and influential factors of cardiometabolic disease prevalence with respect to 230 administrative districts in South Korea. Global Moran’s I was calculated to determine whether the standardized prevalences of cardiometabolic diseases (hypertension, stroke, and diabetes mellitus) were spatially clustered. The CART algorithm was then applied to generate decision tree models that could extract the diseases’ regional influential factors from among 101 demographic, economic, and public health data variables. Finally, the accuracies of the resulting model–hypertension (67.4%), stroke (62.2%), and diabetes mellitus (56.5%)–were assessed by ten-fold cross-validation. Marriage rate was the main determinant of geographic variation in hypertension and stroke prevalence, which has the possibility that married life could have positive effects in lowering disease risks. Additionally, stress-related variables were extracted as factors positively associated with hypertension and stroke. In the opposite way, the wealth status of a region was found to have an influence on the prevalences of stroke and diabetes mellitus. This study suggested a framework for provision of novel insights into the regional characteristics of diseases and the corresponding influential factors. The results of the study are anticipated to provide valuable information for public health practitioners’ cost-effective disease management and to facilitate primary intervention and mitigation efforts in response to regional disease outbreaks.

## Introduction

The geographical variations and influential factors of diseases have been intensively studied in recent years [1–12]. Although recent studies dealt with various kinds of diseases on different scales (i.e. international, national, regional, and local), the common main purpose has been the investigation of the behaviors, conditions, and/or exposures that decisively influence disease incidence or prevalence [13]. Providing reliable and timely information related to disease outbreaks, these studies have the potential to be utilized in augmenting existing etiologic hypotheses and finding undiscovered casual chains in the pathogenesis of diseases, thereby helping to effectively accomplish primary prevention or mitigation of diseases in the public health field [14]. Certainly, epidemiologists, public health practitioners, and medical researchers can refer to this knowledge when initiating regional health promotion programs, prioritizing appropriate treatments specifically required in their communities, and concentrating resources for evidence-based interventions.

For identification of the epidemiologic characteristics of diseases and their corresponding influential factors at the regional level, geographic information systems (GIS) is one of the most powerful tools [15]. Among the various GIS techniques, spatial autocorrelation analysis enables understanding of the characteristics of regional disease statuses. For example, the prevalence pattern of a disease that indicates a significant ‘spatial’ dependency could have different geographical characteristics from those of other diseases that indicate spatially ‘random’ distributions. Based on the clues derived from GIS analytics, data-mining techniques have the potential to discover latent and unexpected mechanisms of disease outbreaks from vast medical and clinical data, which mechanisms are difficult to identify solely by human insight [16]. Therefore, combining GIS analytics with data-mining algorithm, such as classification algorithm, would lead to principal analytic solutions, particularly in the case of geo-referenced medical data [17]. The output of such an analytic combination is expected to augment influential factor studies by identifying novel dangers to public health.

Several studies have used GIS techniques to understand the spatial variations and trends in disease risk [1, 6–9] or to explore the connections between spatial patterns in diseases and the corresponding risk factors on various geographic scales [2–5, 10–12]. Those studies focused mainly on uncovering the spatial pattern of disease prevalence or incidence using spatial statistics and map visualization [1, 6]. All of them suggested that spatial patterns of diseases could be utilized as supporting evidence for further research on disease outbreak mechanisms. Further, many of those studies endeavored to explain the causes and risk factors of diseases with information derived from their spatial patterns [2–5, 10–12]. Various analytic solutions and statistical methods, moreover, were utilized in exploring potential explanatory variables. However, the previous studies have several limitations. First, most of them investigated only one type of disease, which would not be sufficient for public health practitioners’ comprehensive understanding of disease prevalence and geographic patterns. Second, several of the obtained influential factors were based on only limited numbers and types of variables (e.g. temperature, precipitation, age, sex, poverty indicator, urban accessibility, etc.) that have been well-documented as disease-targeting factors.

To overcome the limitations, this study aimed to obtain comparative data on the geographical distributions of three cardiometabolic diseases, including hypertension, stroke and diabetes mellitus, in South Korea. Also, this study aimed to identify novel influential factors among 101 statistical variables related to demographic, economic, and public health. Since identifying influential factors are also based on ecological level, statistical variables which individually collected, were aggregated by administrative districts.

## Method

### Study area

**Fig 1:**
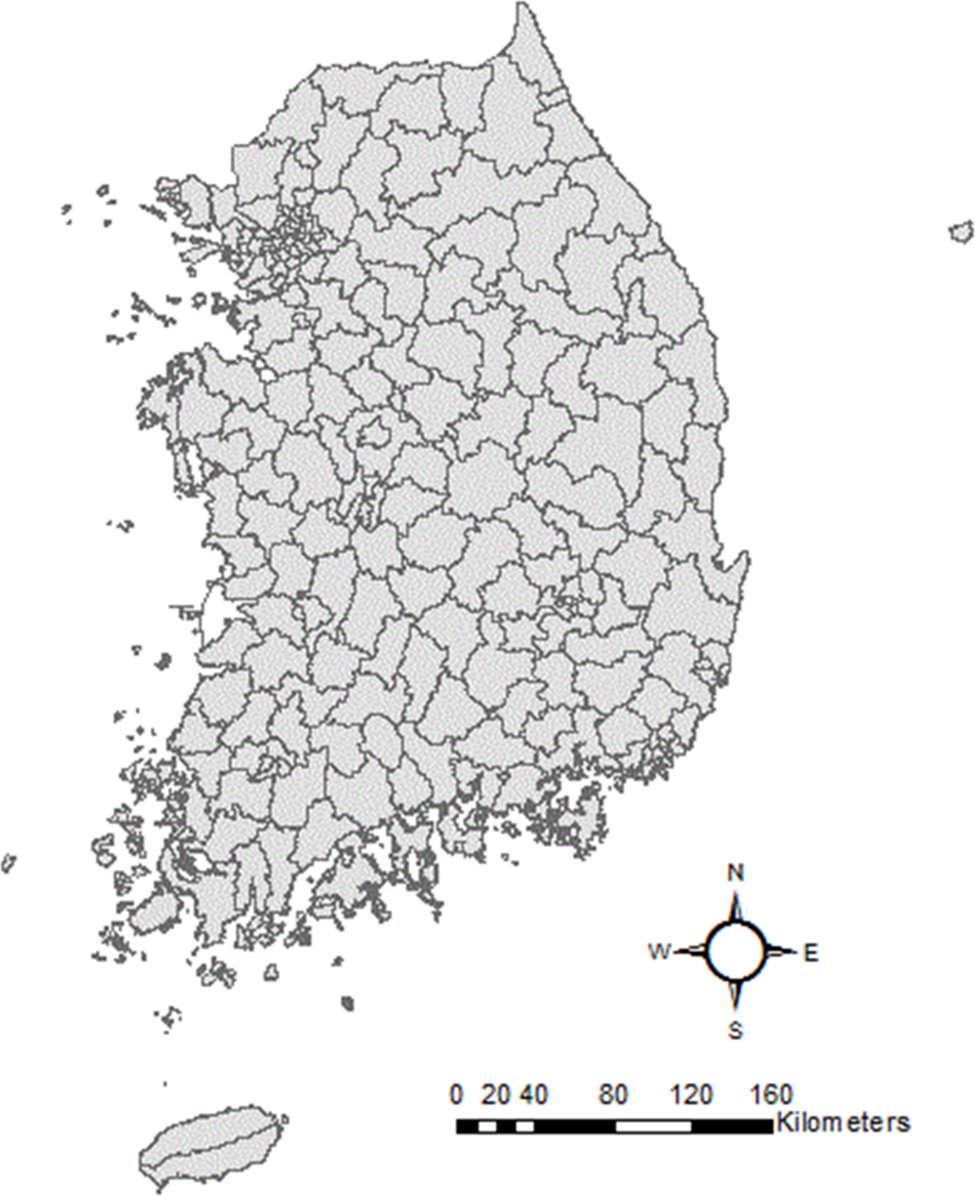
230 administrative districts in South Korea; Source: Statistical Geographical Information Service (SGIS).

The target area of this study comprises 230 administrative districts in South Korea that cover a total area of 99,720 km^2^ (Fig 1). Since this study utilized exhaustively assembled statistical data derived from independent sources, the given administrative districts were determined based on the minimum number of regional units of geographical datasets. The administrative district map was acquired from Statistical Geographical Information Service (SGIS) [18].

### Target variables

To estimate the representative health-related indicators in South Korea, the Korean Centers for Disease Control (KCDC) have conducted Korean Community Health Surveys (KCHS) and have provided the derived data to the public annually since 2008. KCHS, the most representative public health survey in South Korea, is highly valued for its community-based, cross-sectional approach entailing inspection via direct, on-site interviews by trained interviewers. As such, it can obtain detailed information on immunizations, morbidity, health care utilization, disease states, and so forth. Before the present survey was conducted, a sampling frame was designed in combination with the following information: the national address data provided by the Ministry of Public Administration and Security, and the housing-type and number-of-household-member data provided by the Ministry of Land, Transport and Maritime Affairs. From this information, a national representative household sample representing an average of 900 adults aged 19 and over per administrative district was extracted for interviews. Accordingly, a total of 228,921 people were surveyed in 2012. This survey data was classified into Korean administrative-district units called ‘Si-Gun-Gu’. The age- and sex-adjusted disease (hypertension, diabetes mellitus and stroke) prevalences classified into three, tertile-based categories—low, medium, and high prevalence—were used as the ‘target variables’ [19–22].

### Explanatory variables

The Korean Statistical Information Service (KOSIS) has offered to the public various types of cross-sectional statistical data (e.g. population, employment, economy, finance, health, education, etc.) on each administrative district since 2006 [23]. In this study, 101 statistics measured in 2012 were acquired from KOSIS and KCHS to cover all possible data that can be used as potential ‘explanatory variables’ for disease prevalence; further, they were collated, with the target variables, by district unit. The explanatory variables comprised 13 Economic factors, 17 Demographic factors, and 71 Public health variables (S1 Table). Economic factors consist of various tax categories that can be regarded as a region’s wealth indicators. Demographic factors cover population movement, marriage-related statistics, and birthrates. The public health variables were collated from KCDC, and the individual health indicators were aggregated with respect to the 230 administrative districts. EuroQol Five Dimension Questionnaire (EQ-5D) results were included as public health variables. The explanatory variables were standardized to a range from 0 to 1 in order to enable comparison of differently scaled data [24].

### Spatial autocorrelation

Spatial autocorrelation can be utilized in geo-referenced data analysis where the values of an entity at a specified spatial location depend on its values at an adjacent location [25]. For example, the pattern of disease prevalence that indicates significant ‘spatial’ dependency could be different from that of disease prevalence with a spatially ‘random’ distribution. In our study, Moran’s I, a global measure for spatial autocorrelation, was used to identify the spatial dependency of disease prevalence within districts. Moran’s I is defined as

$$I=\frac{N\sum_{i}^{N}\sum_{j}^{N}W_{i.j}(X_{i}-\bar{X})(X_{j}-\bar{X})}{(\sum_{i}^{N}\sum_{j}^{N}W_{i,j})\sum_{i}^{N}(X_{i}-\bar{X}{)}^{2}}$$ (1)

where *N* is the number of observations; *X*_*i*_,*X*_*j*_ are the variable values at *i* and *j*; $\bar{X}$ is the mean of the variables; *W*_*i*,*j*_ is a weight matrix between location *i* and *j*. In this study, the inverse distance squared method was selected to define the weight matrix.

As an extension of the Pearson product-moment correlation coefficient, Moran’s I value ranges from -1 to +1. A value close to 0 indicates a spatially random distribution of variables; a value close to +1 indicates a clustered distribution, and a value close to -1 indicates a dispersed distribution [26]. The z-score is calculated to determine the statistical significance of a Moran’s I value [27]. In this study, a significance level of 0.05 was used. The Z-score is defined as

$$z=\frac{I-E\left\{I\right\}}{\sqrt{Var\{I\}}}$$ (2)

where *E*{*I*} is the expected value of Moran's I, and *Var*{*I*} is its variance.

### Decision tree analysis

In this study, the CART algorithm was implemented to determine the latent associations between regional disease prevalence and 101 statistical variables using the RPART package provided in R. This algorithm has its advantages: it can extract key variables among a myriad of potential explanatory variables, and it can also provide an intuitive and self-exploratory model for the decision-making process. Moreover, the extracted variables can be interpreted as regional characteristics or influential factors associated with the prevalence of the target disease.

#### Decision tree and pruning algorithm

The first stage is to determine classification rules for generating a decision tree. The tree is built by a recursive partitioning process. A variable that best splits the data into two groups with maximum homogeneity is determined among all explanatory variables based on the impurity function. In this study, the Gini index, which, with Information Gain, is the most commonly selected for classification, was chosen as the splitting criterion [28]. The Gini index utilizes the impurity function

$$gini(T)=1-\sum_{i\neq j}^{J}p(i|T)p(j|T)$$ (3)

where *T* is the given dataset; *i* and *j* are classes in dataset *T*, *J* is the number of classes in *T*; *p*(*i*|*T*) is conditional probability of class in *i* dataset *T*.

Implementing the impurity function, the CART algorithm searches variables and their corresponding splitting values within all explanatory variables that maximize the following impurity change in all partitioning procedures:

$$Gini_{split}(T)=-gini(T)+\frac{N_{L}}{N}gini(T_{L})+\frac{N_{R}}{N}gini(T_{R})$$ (4)

where *T* is the given dataset; *T*_*L*_ and *T*_*R*_ are datasets of left and right child of *T*_*L*_ respectively; *T*_*L*_ is the number of tuples in *T*; *N*_*L*_ and *N*_*R*_ are the number of tuples in *T*_*L*_ and *T*_*R*_ respectively.

After selecting best variable and corresponding value and generating two sub-groups (child datasets), this process is implemented for each sub-group, and so on recursively, until the terminal nodes contain only one class. The final model consists of three components: the root node, internal nodes, and leaf nodes. The root node, the topmost node in the tree, can be regarded as the most influential factor to explain the given entire dataset, while its branching child nodes (internal nodes) explain well what follows behind. Finally, the leaf nodes represent the final categories to which the classification model assigns the original dataset. The second decision tree stage is to build an optimal size of tree using a pruning algorithm. The tree, at its maximal growth, can be highly complex, offering only poor classification performance (the so-called over-fitting problem), and its myriad of decision nodes can render it unintelligible. Therefore, pruning is demanded in order to give decision models validity and to improve comprehensibility. In this study, ten-fold cross-validation was used, not only to select the best-pruned tree offering the best validation accuracy but also to estimate the future classification accuracy of a decision model from the given past dataset [29].

#### Accuracy assessment and interpretation of model

**Fig 2:**
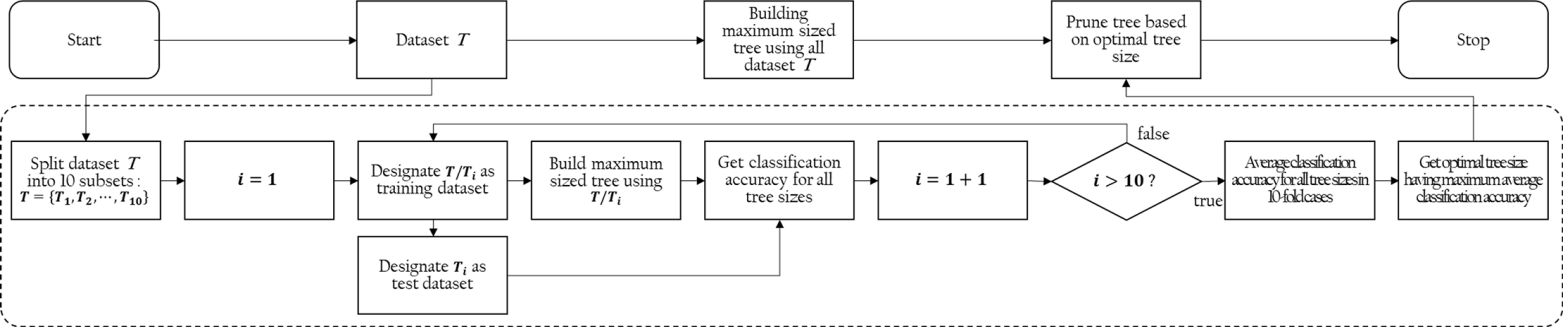
Flow chart for generation of optimally pruned tree with maximum classification accuracy based on ten-fold cross-validation. The optimal tree size is determined from the point where the average classification accuracy in the 10-fold cases is maximized.

Fig 2 is a flow chart illustrating the procedure for generation of an optimally pruned tree with maximum classification accuracy based on ten-fold cross-validation. First, the fully grown tree is generated using the entire dataset *T* and denoted as the ‘final model’. Then, dataset *T* is randomly partitioned into 10 subsets. In the first loop, 9 out of 10 subsets, denoted as the training dataset, are used to generate another tree, and the last 1 subset, denoted as the test dataset, is used to calculate the validation accuracy for all possible tree sizes given the tree model. This process is repeated 10 times for each subsets, and the average classification accuracy with respect to the tree sizes is reported. Finally, the optimal tree size is determined from the point where the average classification accuracy becomes maximized. The final model is then pruned according to the optimal tree size, and the average classification accuracy in the optimal tree size is taken as the model accuracy.

The decision nodes resulting from the analysis are the best explanatory variables among the given 101 statistical variables. The CART algorithm allocates each node based on the following rule: regions that are assigned to left-child nodes by the classification rule from a parent node have a lower prevalence than the ones that are assigned to right-child nodes. This means that explanatory variables in parent nodes can be classified into positive influential factors (variables of which the higher standardized value yields higher prevalence), and negative influential factors (variables of which the lower standardized value yields higher prevalence). Moreover, the classification rules at the lower tree depth tend to have more influence on the national-scale prevalence than those at the higher depth. This is due to the fact that those rules selected as the root node, which has the lowest depth, classify with all administrative districts, whereas the rules at the higher tree-depth classify only with a limited number of regions that meet the classification rules of their parent nodes.

## Results

### Spatial dependency

**Fig 3:**
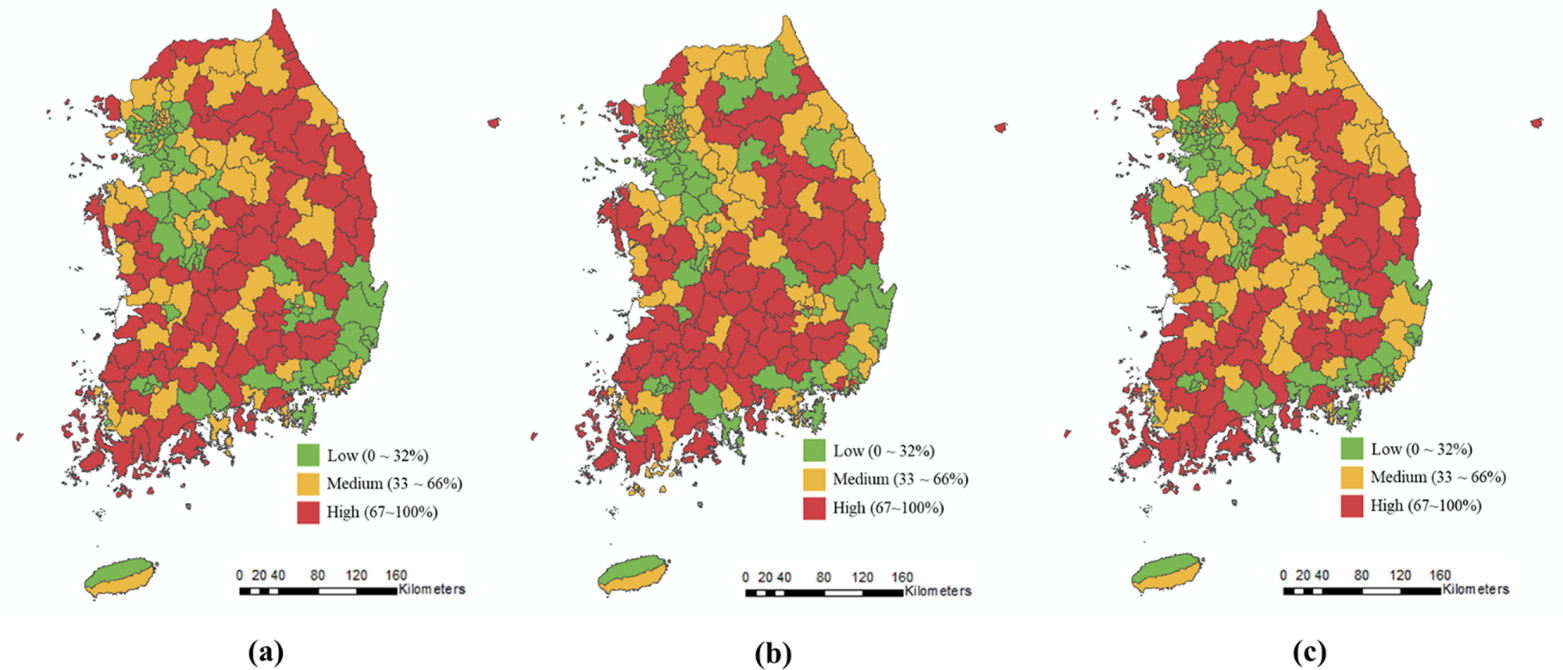
**Spatial distribution of three cardiometabolic diseases:** (a) Hypertension; (b) Stroke; (c) Diabetes mellitus; Portions of this document/figure include intellectual property of Esri and its licensors and are used under license. Copyright [31, Aug., 2018.] Esri and its licensors. All rights reserved.

Table 1 shows the results of Moran’s I calculation and its statistical significances for the three cardiometabolic diseases. All of the diseases showed the existence of spatial autocorrelation with the significance level of 0.01. Hypertension (I = 0.30) showed the highest positive Moran’s I value, followed by Diabetes mellitus (I = 0.26) and Stroke (I = 0.24). Fig 3(A)–3(C) show the diseases’ choropleth maps. The distinctive spatial patterns and correspondents to high Moran’s I values indicate that each disease turns upon certain geographic or environmental factors in peculiar ways.

**Table 1:** Statistical test of Moran's I for each disease.

Disease	Moran’ I	z-score
Hypertension	0.30	5.69
Stroke	0.24	4.47
Diabetes mellitus	0.26	4.96

Fig 3(A) illustrates the spatial distribution of hypertension. Low prevalence was clustered in the Seoul capital as well as the southeastern coastal are (especially the Busan metropolitan area), while high prevalence was clustered across the central area. Fig 3(B) depicts the spatial distribution of stroke. Low prevalence was clustered around the Seoul capital area and in the southeastern coastal area, while high prevalence was clustered across the central eastern and southwestern areas. Fig 3(C) illustrates the spatial distribution of diabetes mellitus. Low prevalence was clustered in the Seoul capital area and in the southeastern coastal area, while high prevalence was clustered across the central area.

### Diagnostics of regional disease prevalence

**Fig 4:**
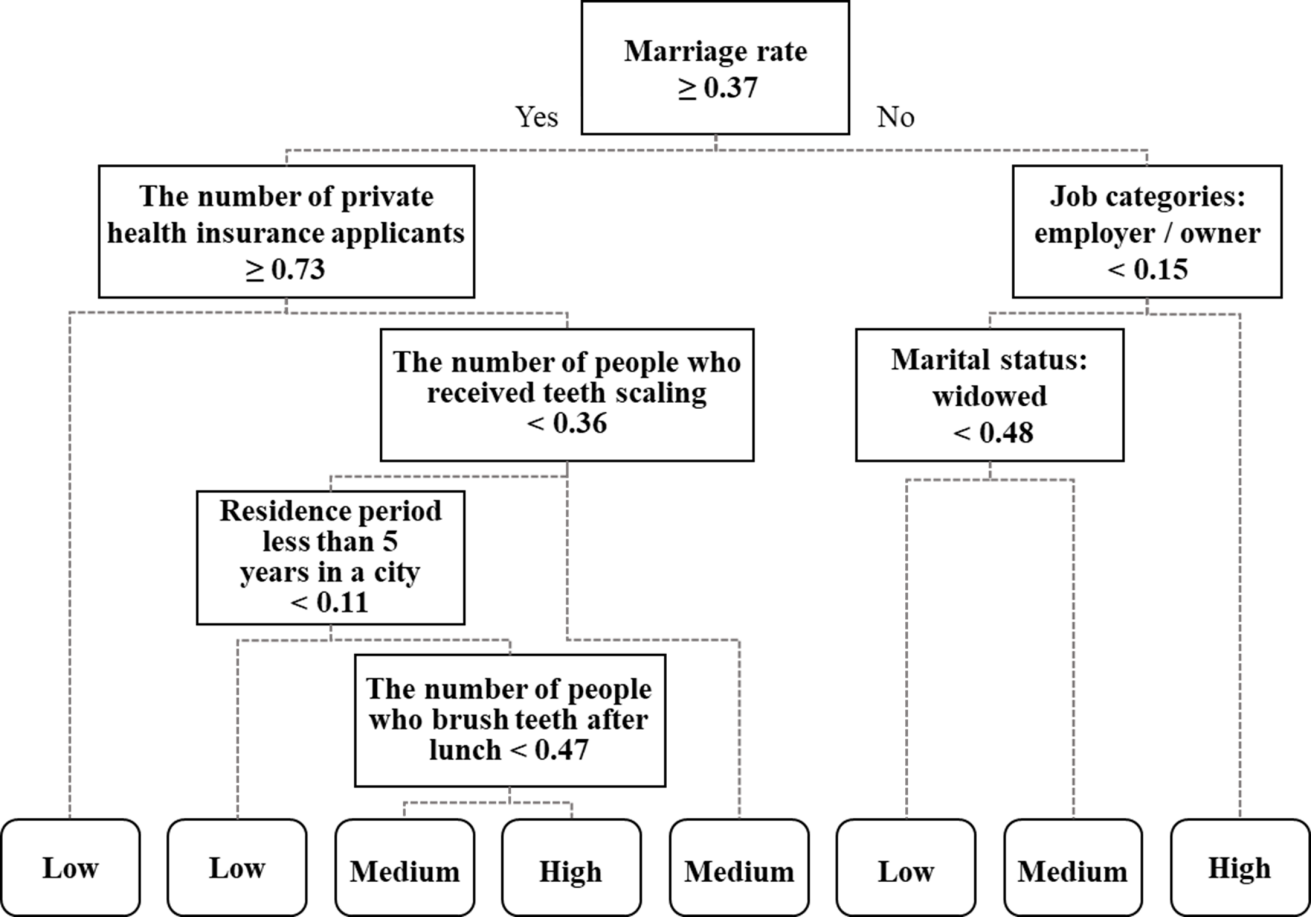
Influential factors of hypertension extracted from decision tree model.

**Fig 5:**
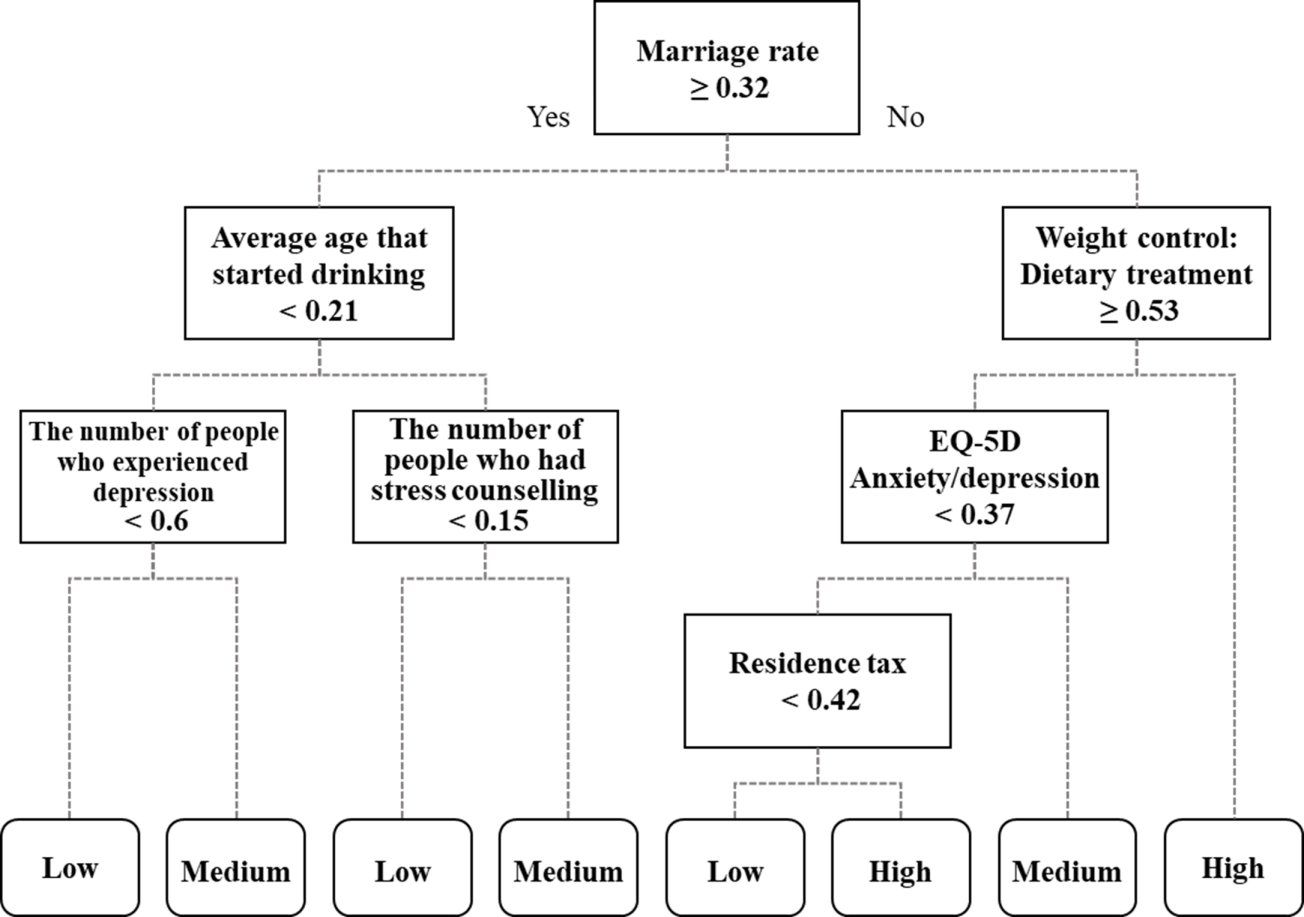
Influential factors of stroke extracted from decision tree model.

**Fig 6:**
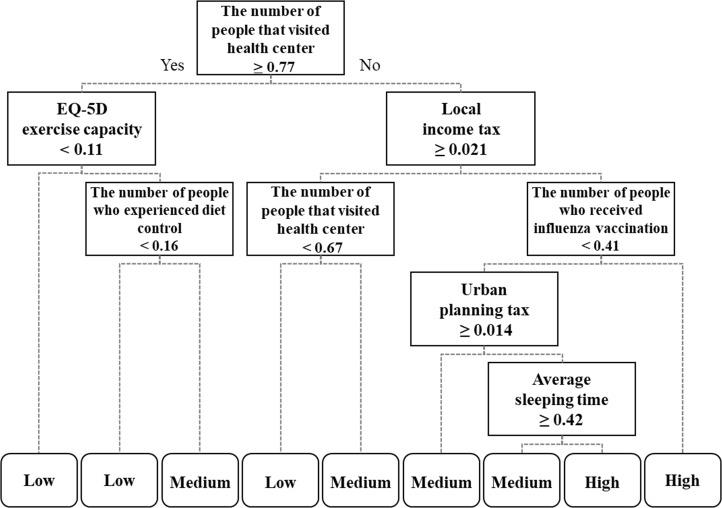
Influential factors of diabetes mellitus extracted from decision tree model.

Decision tree models for the given three diseases were generated using CART and the pruning algorithm with 101 statistic data as the ‘explanatory variables’ and each disease prevalence level–low, medium, high–as the ‘target variables’. Figs 4–6 demonstrate the decision tree results. As a result of ten-fold cross-validation for accuracy assessment, the tree model of hypertension presented the highest classification accuracy (67.4%), followed by stroke (62.2%) and diabetes mellitus (56.5%). The classification models showing such accuracy were assumed to be satisfactory and meaningful, in that 5% of the 101 potential explanatory variables could classify the three disease prevalences. Additionally, the spatial distributions along with the positive and negative influential factors for the three diseases are provided in Table 2. The positive influential factors indicate variables of which the higher standardized value yields higher prevalence, while the negative influential factors indicate variables of which the lower standardized value yields higher prevalence. The influential factors for the three disease prevalences were analyzed in more detail as follows.

**Table 2:** Spatial distribution with positive and negative influential factors for three cardiometabolic diseases.

Disease (Accuracy)	Spatial distribution	Positive influential factors	Negative influential factors
Hypertension (67.4%)	• **Low prevalence is clustered in Seoul capital area and in southeastern coastal area, while high prevalence is clustered across central area.**	• **Job categories: employer / owner** • **Number of people who received teeth scaling** • **Marital status: widowed** • **Residence period less than 5 years in city** • **Number of people who brush teeth after lunch**	• **Marriage rate**^a^ • **Number of private health insurance applicants**
Stroke (62.2%)	• **Low prevalence is clustered around Seoul capital area and southeastern coastal area, while high prevalence is clustered across central eastern and southwestern areas.**	• **Average age started drinking** • **Number of people who experienced depression** • **Number of people who had stress counseling** • **EQ-5D Anxiety/depression** • **Residence tax**	• **Marriage rate**^a^ • **Weight control: Dietary treatment**
Diabetes mellitus (56.5%)	• **Low prevalence rate is clustered in Seoul capital area and in southeastern coastal area, while high prevalence rate is clustered across central area.**	• **EQ-5D exercise capacity** • **Number of people who exercise diet control** • **Number of people who received influenza vaccination**	• **Number of people who visited health center**^**a**^ • **Local income tax** • **Urban planning tax** • **Average sleeping time**

^a^ indicates attributes selected as root node. The positive influential factors indicate variables of which the higher standardized value yields higher prevalence, while the negative influential factors indicate variables of which the lower standardized value yields higher prevalence.

#### Hypertension

The influential factors for hypertension were extracted from the decision tree model as depicted in Fig 4: ‘Job categories: employer / owner’, ‘Number of people who received teeth scaling’, ‘Marital status: widowed’, ‘Residence period less than 5 years in a city’, and ‘Number of people who brush teeth after lunch’ were extracted as positive influential factors. A higher number for ‘Job categories: employer / owner’ in a region (≥ 0.15) showed a higher prevalence of hypertension. In addition, a higher level of dental hygiene, which was represented as ‘Number of people who received teeth scaling’ (≥ 0.36) and ‘Number of people who brush teeth after lunch’ (≥ 0.47) yielded a higher prevalence. Moreover, regions with larger numbers of people with ‘Marital status: widowed’ (≥ 0.48) showed higher hypertension prevalence. Finally, regions with more people with ‘residence period less than 5 years in a city’ (≥ 0.11) showed higher hypertension prevalence. Also, there were negative influential factors of hypertension incidence. For example, regions with lower ‘Marriage rate’ (< 0.37) showed higher prevalence of hypertension. Also, regions with smaller values for ‘Number of private health insurance applicants’ (< 0.73) showed higher prevalence of hypertension.

#### Stroke

Fig 5 shows the resulting decision tree with the indicated novel influential factors for stroke. ‘Average age started drinking’, ‘Number of people who experienced depression’, ‘Number of people who had stress counseling’, ‘EQ-5D anxiety/depression’, and ‘Residence tax’ were extracted as positive influential factors of stroke. In detail, regions with higher ‘Average age that started drinking’ (≥ 0.21) had a higher stroke prevalence. Additionally, higher levels of depression and stress, represented by ‘Number of people who experienced depression’ (≥ 0.6), ‘Number of people who had stress counseling’ (≥ 0.15), and ‘EQ-5D Anxiety/depression’ (≥ 0.37), were correlated with higher stroke prevalence. Moreover, regions paying more ‘Residence tax’ (≥ 0.42) showed higher stroke prevalence as well. In contrast, ‘Marriage rate’ and ‘Weight control: dietary treatment’ were extracted as negative influential factors of stroke prevalence. Similar to the case of hypertension, regions with lower ‘Marriage rate’ (< 0.32) were found to have higher stroke prevalence. Finally, higher prevalences of stroke were found in regions with more people that had experienced ‘weight control: Dietary treatment’ (< 0.53).

#### Diabetes mellitus

The corresponding decision tree is depicted in Fig 6. According to it, ‘EQ-5D exercise capacity’, ‘Number of people who experienced diet control’, and ‘Number of people who received influenza vaccination’ were extracted as positive influential factors. To be specific, regions with a higher score for ‘EQ-5D exercise capacity’ (≥ 0.11) showed a higher prevalence in diabetes mellitus. Additionally, regions having more ‘people who experienced diet control’ (≥ 0.16) were found to show higher prevalence in diabetes mellitus. Moreover, the more ‘people there were who received influenza vaccination’ (≥ 0.41), the higher was the prevalence rate that was shown. As for the negative influential factors for diabetes mellitus, higher prevalence in diabetes mellitus was found in regions with fewer ‘people that visited health center’ (< 0.77). Regions paying less ‘Local income tax’ (< 0.021) or ‘Urban planning tax’ (< 0.014) were also found to have higher prevalences of diabetes mellitus. Finally, the shorter the ‘Average sleeping time’ (< 0.42) was, the higher was the prevalence of diabetes mellitus.

## Discussion

In the present study, we attempted to explore the geographical variations and influential factors for hypertension, stroke, and diabetes mellitus in 230 administrative districts in South Korea. As a result of spatial autocorrelation analysis, all three diseases showed statistically significant spatial autocorrelation. Then, decision tree models of each disease were generated using CART and a pruning algorithm. After assessing model accuracy with ten-fold cross-validation, positive and negative influential factors of the diseases were presented, and some important insights were derived from factor analysis. However, there are some issues conducting statistical analysis of geographical data. Classical problem called modifiable areal unit problem (MAUP) which significantly impacts the result, should be considered. The MAUP was first identified by [30]. Its idea is that, the statistical results using same basic data in the same study area can be different when the study area is aggregated in different ways. However, in this study we only focused on the determination of influence factors based on 230 administrative districts in South Korea.

The results of the factor analysis for the three cardiometabolic diseases suggested that marriage rate, which was selected as the root node in the tree models of hypertension and stroke, was a negative influential factor for those diseases. The fact that married people showed lower prevalence of diseases might imply that married life has positive effects on the reduction of risks of hypertension and stroke incidence. Some influential factors were unique for each cardiometabolic disease. In the case of hypertension, regions with more people who experienced bereavement showed higher risks of hypertension incidence. The findings of this study corroborate the results from previous studies regarding the common predictors, including marital status, depression, and sleep duration [31]. Never-married men had a higher risk of hypertension relative to those who were married. In another recent study, marital history was also significantly associated with survival after stroke [32]. Compared with those who were married, the risk of dying following a stroke was significantly higher among never-married men or widowers.

Additionally, stress-related factors were also positively associated with prevalence of hypertension and stroke. For example, ‘Job categories: employer / owner’, one of the positive influential factors of hypertension, suggested that independent business owners’ stress could be one of the causes for hypertension incidence. Similarly, the greater the number of people who experienced depression or had stress counseling, the higher the prevalence of stroke that was shown. Moreover, the results showed that the wealth status of the region had the opposite influence on prevalence of stroke and diabetes mellitus. For example, residence tax, which is imposed in proportion to one’s income, was found to be a positive influential factor for stroke prevalence, thereby indicating that higher-income classes show higher prevalences of stroke. On the other hand, local income tax and urban planning tax, which are imposed according to one’s income level and land (housing and buildings) owned, respectively, were found to be negative influential factors for diabetes mellitus, thereby indicating that prevalence was higher in lower-wealth-status regions.

Regions providing high levels of health care services were found to have low risk in the prevalence of hypertension. In the case of stroke, dietary treatment operated as a means to decrease stroke prevalence, since weight control and dietary treatment showed negative relations with stroke prevalence. In the case of diabetes mellitus, the average sleeping duration showed a negative relation with the diabetes prevalence rate. Finally, the more people visited a health center, the lower the prevalence of diabetes mellitus was.

Several studies have reported risk factors for prevalence of cardiovascular disease at the community level, which factors have not been fully accounted for at the individual level [33–35]. For example, regional-based measures of socioeconomic status, which are represented as income adequacy, household income, migration rate, and accessibility to health care resources, are found to have relationships with high cardiovascular disease prevalence. Those results can be considered to be supporting evidence validating influential factors derived from decision tree models. On the other hand, in individual-level data analysis [36–38], it has been suggested that depression, stress and sleep duration are associated with high prevalences of stroke and diabetes mellitus respectively, which conclusions correspond with the results of this study. In a dose-response meta-analysis of prospective studies, a U-shaped relationship between sleep duration and risk of type 2 diabetes was shown [31].

It was also interesting to find out that as people are more aware of dental hygiene, the prevalence in hypertension increases. The relationship between dental hygiene and hypertension prevalence has not been fully clarified yet, and remains to be evaluated. Poor oral hygiene, exemplified by high levels of dental plaque and dental calculus, among other conditions, also was associated with risk of hypertension [32]. Other studies, however, have found conflicting results on the association between dental hygiene and hypertension. Tooth scaling for example was associated with decreased risk of future cardiovascular events such as myocardial infarction, stroke, and all cardiovascular events [39].

## Conclusion

This study highlights significances in four perspectives. First, this study provided comparative results on the geographical distributions of three different diseases in 230 administrative districts in South Korea. Second, geographic properties were considered in classifying the tertile prevalence groups of the given diseases and in identifying corresponding influential regional factors. Third, statistical data was exhaustively collated from the most representative, highly regarded community-based and cross-sectional public health survey in South Korea. Finally, data-mining techniques were utilized to identify the latent and underlying influential factors of cardiometabolic diseases, avoiding bias from the well-documented knowledge about the diseases.

There are several limitations to this study that merit further investigation. First, the process implemented in this study is static in time. From the perspective of disease monitoring, time variance is a crucial property, since geographical factors and disease patterns change over time. In future work therefore, an identical framework will be applied to data from different years. Second, the scales of the 230 administrative districts vary significantly: a metropolitan region has a smaller spatial unit, and rural region has a larger one. Therefore in the future study, the statistical analysis should be conducted in various scale and aggregation basis. By differentiating the scale and aggregation method, analyzing the influential factors of diverse diseases can be specific and efficient. Finally, in-depth and further investigation into influential disease factors from the perspectives of epidemiology and pathogenesis also is required.

This study suggested, a framework that not only shows that regional characteristics are closely associated with the disease status of that region but also provides novel and unexpected insights, particularly as the potential explanatory variables were exhaustively assembled without incurring any bias from the well-documented knowledge on the three diseases investigated. The results of this study, therefore, are anticipated to provide valuable information to public health practitioners’ cost-effective disease management and to facilitate primary intervention and mitigation efforts in response to regional disease outbreaks.

## Supporting information

S1 Table Statistic dataset exhaustively collated from Korean Statistical Information Service (KOSIS) and Korean Community Health Survey (KCHS). (DOCX)
