# Supplementary material for: Geographical variations and influential factors in prevalence of cardiometabolic diseases in South Korea
Source: PLoS One. 2018 Oct 2;13(10):e0205005. doi: 10.1371/journal.pone.0205005 (PMC6168158; doi:10.1371/journal.pone.0205005)
Supplement: S1 Table — (DOCX) [file pone.0205005.s001.docx]

# Supporting information

**S1 Table. Statistic dataset exhaustively collated from Korean Statistical Information Service (KOSIS) and Korean Community Health Survey (KCHS)^a^**

| Category (n) | Explanatory variables | | |
| --- | --- | --- | --- |
| Economic factors  (13) | - Total local tax - Acquisition tax - Automobile tax - Regional development tax - Urban planning tax | - Local education tax - License tax - Property tax - Revenue tax for previous year - Local income tax | - Residence tax - Monthly income - Number of economically  active persons |
| Demographic factors  (17) | - Job categories: employer / owner - Job categories: employee - Job categories: unpaid family worker - Marital status: live together - Marital status: separated - Marital status: widowed - Marital status: divorced | - Residence period less than  5 years in a city - Residence period less than  5~10 years in a city - Residence period less than 10~15 years in a city - Residence period less than 15~20 years in a city | - Residence period more than 20 years in a city - Marriage rate - Birthrate - Divorce rate - Average height - Average weight |
| Public health variables  (71) | - Number of people living with dementia - Number of people who smoked - Number of current smokers - Number of people who are drunk - Number of people that drinks alcohol - Number of people who sought counseling about drinking problem - Number of people who participated in exercise programs in a community - Weight control: exercise - Weight control: fasting - Weight control: dietary treatment - Weight control: diet pills with no prescription - Weight control: diet pills with prescription - Number of people who brush teeth after lunch - Number of people who neglected mandatory dental treatment - Number of people with experience of oral treatment - Number of people who received teeth scaling - Number of people who had stress counseling - Number of people who experienced depression - Number of people who had counseling about depression - Number of people who received influenza vaccination - Number of people who experienced health checkup - Number of people who experienced acute disease and/or addiction to | - Number of people who received medical services - Number of people who experienced damage cause - Number of people who experienced lying sick in bed - Number of private health insurance applicants - Number of people that visited health center - Average age that started smoking - Average smoking amount per day with everyday smokers - Average smoking days per month with random smokers - Average smoking per day with random smokers - Number of years of smoking _ former smokers - Number of months of smoking _ former smokers - Average smoking amount per day _ former smokers - Average age that started drinking - Drinking frequency - Amount of drinking per day - High-risk drinking rate (Male) - High-risk drinking rate (Female) - Number of people who experienced failure of drinking control - Number of people who experienced difficulties in daily life after drinking - Number of people who experienced black outs after drinking - Number of days with heavy work outs - Hours of heavy work outs | - Average minutes duration of heavy work outs - Number of days with regular work outs - Hours of regular work outs - Average minutes duration of regular work outs - Number of days with walking - Average hours for walking per week - Average minutes for walking per walk - Accessibility to fitness center - Number of days with breakfast in a week - Low salt preference _ average amount of Natrium consumed - Low salt preference _ adding of salt to meals - Low salt preference _ adding of soy sauce to fried meals - Number of people who check nutrient balance - Self-recognition of body shape - Number of people who experienced diet control - Self-rated dental health - Number of people who have difficulties with mastication - Number of people who have difficulties with pronunciation - Average sleeping time - Self-rated stress amount - Self-rated health condition - EQ-5D: mobility - EQ-5D: capacity for self-care - EQ-5D: conduct of usual activities - EQ-5D: pain/discomfort - EQ-5D: anxiety/depression - EQ VAS: current health status |

^a^Available at <http://​www.​kosis.​kr> and https://chs.cdc.go.kr/chs/index.do
